# FACTORS ASSOCIATED WITH LENGTH OF HOSPICE STAY

**DOI:** 10.1093/geroni/igz038.886

**Published:** 2019-11-08

**Authors:** Megumi Inoue, Matthew G Kestenbaum, Cameron Muir

**Affiliations:** 1 George Mason University, Fairfax, Virginia, United States; 2 Capital Caring, Falls Church, Virginia, United States; 3 Capital Caring, Washington DC, District of Columbia, United States

## Abstract

The benefits of early referral to hospice services have been well documented. However, late admissions and short hospice stays are ongoing issues that are often barriers to improving terminally-ill persons’ and the caregivers’ quality of life and the quality of care. Using ordered logistic regression, this study analyzed the length of stay of 7,307 patients who died in 2015 while receiving care in the largest hospice agency in the DC metro region. Cancer diagnoses and residence in a higher median income neighborhood were associated with shorter lengths of stay. Female sex, older age, and residence in a lower median income neighborhood were associated with longer lengths of stay. The findings indicate that differences in demographic and diagnostic characteristics likely affect hospice length of stay.

